# Field performance of three mosquito collection methods for assessing the entomological efficacy of dual-active ingredient long-lasting insecticidal nets

**DOI:** 10.1038/s41598-023-39558-9

**Published:** 2023-07-28

**Authors:** Boulais Yovogan, Constantin J. Adoha, Bruno Akinro, Manfred Accrombessi, Edouard Dangbénon, Aboubakar Sidick, Razaki Ossè, Gil G. Padonou, Louisa A. Messenger, Arsène Fassinou, Hermann W. Sagbohan, Clément Agbangla, Armel Djènontin, Esdras M. Odjo, Corine Ngufor, Jackie Cook, Natacha Protopopoff, Arthur Sovi, Martin C. Akogbéto

**Affiliations:** 1 Faculté des Sciences et Techniques, Université d’Abomey-Calavi, Abomey-Calavi, Benin; 2 Centre de Recherche Entomologique de Cotonou, Cotonou, Benin; 3 Faculty of Infectious and Tropical Diseases, Department of Disease Control, London School of Hygiene and Tropical Medicine, London, UK; 4 Ecole de Gestion et d’Exploitation des Systèmes d’Elevage, Université Nationale d’Agriculture, Kétou, Benin; 5 Department of Environmental and Occupational Health, School of Public Health, University of Nevada, Las Vegas, NV 89154 USA; 6 Medical Research Council (MRC) International Statistics and Epidemiology, Epidemiology Group, London School of Hygiene and Tropical Medicine, London, UK; 7 Faculté d’Agronomie, Université de Parakou, Parakou, Benin

**Keywords:** Zoology, Diseases

## Abstract

Selection of mosquito collection methods is of crucial importance to evaluate the impact of vector control tools on entomological outcomes. During a cluster randomised control trial evaluating the relative efficacy of two dual-active ingredient (a.i.) long-lasting insecticidal nets (LLINs) compared to pyrethroid-only LLINs, we assessed the performance of different mosquito collection methods: Human landing catches (HLC), Centers for Disease Control and Prevention (CDC) light traps, and pyrethrum spray catches (PSC). *Anopheles* mosquitoes were collected using three collection methods in 4 houses, in each of the 60 trial clusters at baseline and every quarter for 24 months using PSCs and HLCs, while CDC light traps were performed during two quarters only. Mean density of vectors collected per method per night was the highest with HLCs (15.9), followed by CDC light traps (6.8); with PSCs (1.1) collecting 10 times less mosquitoes than HLCs. All three collection methods collected fewer mosquitoes in the Interceptor G2^®^ dual a.i. arm, compared to the other trial arms, although only HLCs and PSCs demonstrated strong evidence of this due to a greater number of collection rounds undertaken, than CDC light traps. The broadly similar results regarding the differential impact of the two dual a.i. LLINs showed by the three collection methods suggest that the more ethically acceptable, cheaper, and logistically simpler methods such as CDC light traps could be prioritised for use in large community trials for measuring the efficacy of vector control tools.

## Introduction

Long-lasting insecticidal nets (LLINs) and indoor residual spraying (IRS) remain the two pillars on which malaria vector control relies globally^1^. To assess the impact of these tools on key entomological and malaria transmission indicators such as malaria vector species density, composition, *Plasmodium* sporozoite rate (SR) and entomological inoculation rate (EIR), the collection method used for systematically collecting mosquito vectors is key.

Human landing catches (HLC) involve the collection of mosquitoes from the legs of a collector, using a flashlight and a manual aspirator. HLCs are considered the gold standard method given that it provides a direct measurement of the frequency of person-vector contact^2^. However, this collection method raises ethical concerns, as it can lead to increased exposure of collectors to infected mosquito bites. Although mosquito collectors can be protected with malaria chemoprophylaxis^3,4^, the risk of being infected by other mosquito-borne diseases, such as arboviruses, remains. In addition, this collection method requires intense overnight supervision to ensure the reliability of the data collected, and is strongly dependent on the skills, level of attractiveness to mosquitoes, and experience of the collectors. HLCs are therefore labour-intensive and expensive, which often means only a limited number of data points can be collected. Given these constraints, alternative mosquito collection methods such as miniature Centers for Disease Control and Prevention (CDC) light traps, and pyrethrum spray catches (PSC) have been evaluated.

The CDC light trap is a simple alternative which is less expensive, easy to standardise and can be deployed at large scale^5^. Originally developed for collecting agricultural pests, CDC light traps are now routinely used to sample *Anopheles* vectors after the works by Odetoyinbo et al*.*^6^ demonstrated their efficacy. This trap usually collects endophilic mosquitoes that have not managed to blood-feed on a host protected by a mosquito net^7^. They use odour cues emitted by the sleeper to attract mosquitoes^8^, and work best when set at the feet of the sleeper^9^. However, due to the presence of light, non-targeted insects may also be collected by this trap^10^. In addition, it may prove ineffective when used alone, or set up in a place where there is a competing ambient light^11^. Also, the unknown response of some mosquito species to light, may lead this trap to provide non-reliable information regarding vector populations^11^.

PSCs involves spreading white sheets over the floor and furniture of houses early in the morning- whilst windows, doors and openings are closed. Aerosol pyrethrum insecticide is sprayed inside the house and after 10–15 min, mosquitoes fallen on the sheets are collected and stored^12^. Likely factors that could influence the efficacy of this collection method include: the behaviour of the house occupants who may open the doors/windows earlier in the morning, thus unconsciously allowing mosquito to escape prior to the collection, and the amount of sprayed insecticide per m^2^ that may not be sufficient to knock down or kill mosquitoes. Apart from that, this collection method may somewhat cause inconvenience to the house occupants as they are asked to go outside and interrupt their daily routines. PSC collects mostly indoor resting mosquitoes^13^.

Previous studies comparing the efficiency of different collection methods reported that CDC light traps and PSCs collected a lower number of *Anopheles* compared to HLCs but that the sporozoite rates in collected *Anopheles* as well as relative species composition were similar^10,14–17^.

In large vector controls trials, the choice of collection method varies, often depending on the acceptability of the method by the country ethics team and/or the size of the trial, with HLCs mainly being used in West African countries^18–20^ and CDC light traps more common in East Africa^21–23^, with some also being used in West Africa^24,25^. Many of the trials supplemented the main collection method with PSCs^26,27^ or Prokopack aspirators to collect data on indoor mosquito resting density^27–29^.

The present study is part of a large three arm cluster randomized controlled trial (RCT) assessing the efficacy of two dual-active ingredient (dual-a.i.) LLINs in Benin on malaria incidence, prevalence and transmission^30^, compared to standard pyrethroid-only LLINs. HLCs were the main entomological collection method used in the RCT. CDC light traps and PSCs were also conducted to explore if they are viable alternatives to HLCs to estimate indoor entomological indicators, and to assess whether these collection methods were as effective as HLCs in evaluating the efficacy of new malaria vector control tools.

## Methods

### Study area

**Figure 1:**
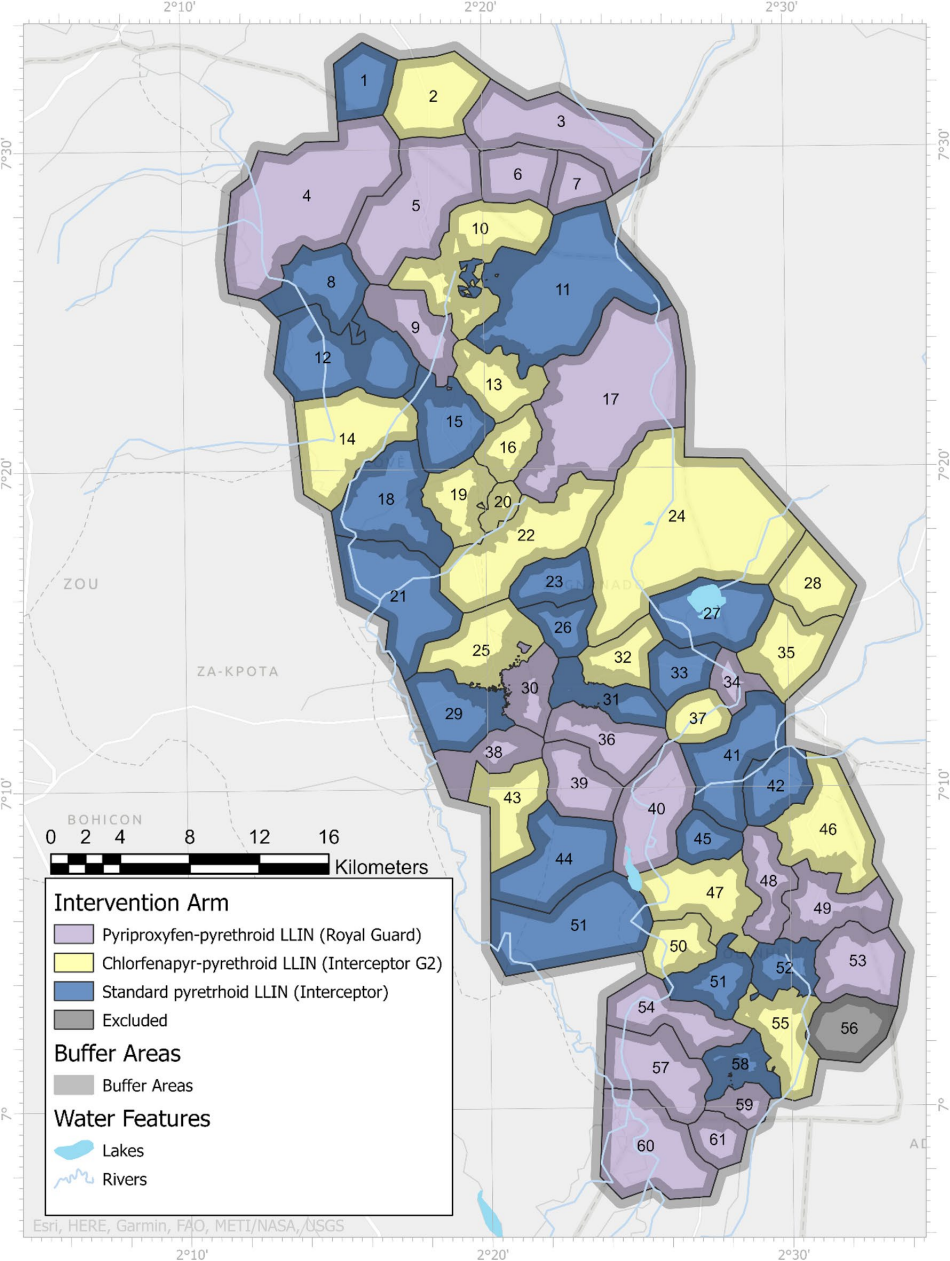
Map of the study area showing the three study arms, each with 20 clusters. The map was produced by study investigators (M.A., E.D., J.C.) with Esri ArcGIS Pro 3.1 software (https://pro.arcgis.com/fr/pro-app/latest/get-started/download-arcgis-pro.htm) using study data and online data provided by GADM (https://gadm.org/download_country.html) and Natural Earth (https://www.naturalearthdata.com/).

The study was conducted in the districts of Covè, Zagnanando, and Ouinhi located in the department of Zou, Southern Benin and is detailed elsewhere^30^. The area has 123 villages and approximately 220,000 inhabitants and was divided into 60 clusters as part of a large RCT. Twenty clusters were randomly allocated to each of the following arms: mixture pyrethroid-chlorfenapyr LLINs: Interceptor^®^ G2 (intervention arm 1), mixture pyrethroid-pyriproxyfen LLINs: Royal Guard^®^ (Intervention arm 2), and pyrethroid-only standard LLINs: Interceptor^®^ (Control arm) (Fig. 1)^20^. Each cluster was composed of core and buffer area of at least 1000 m between households in neighbouring clusters.

A baseline entomological survey in 2019 showed that 95.8% of all *Anopheles* collected were *An. gambiae* s.l. and the remaining were *An. funestus* and *An. nili* groups. In the *An. gambiae* s.l. complex *An. gambiae* s.s. and *An. coluzzi* were present in equal proportions^31^.

Malaria infection prevalence in the population (all ages) was 43.5% prior to the distribution of the nets^32^.

### Mosquito sampling

One data collection round occurred at baseline in September and October 2019 before the net distribution, while 8 post-net distribution rounds were conducted between June 2020 and April 2022. HLC and PSC collections were conducted in all 60 clusters every 3 months. CDC light traps were only conducted at baseline and during two round post-net distribution (16- and 19-months post-net distribution (June-July, and September–October 2021, referred to as Rounds 5 and 6)). For HLC sampling, one house was selected randomly in each cluster, with three further houses selected within 20 m of the randomly selected house (for a total of 4 houses per cluster per round). For PSC sampling, four houses close to the HLC selected houses were used. At baseline, one CDC light trap was placed in a house neighbouring a house used for HLC. The other three houses for CDC light traps were picked randomly in the cluster. Post-net distribution, the CDC light traps were used in houses neighbouring the four houses used for HLCs, similar to the PSC sampling method. There was a total of 240 collection points per round for each collection method.

**Figure 2:**
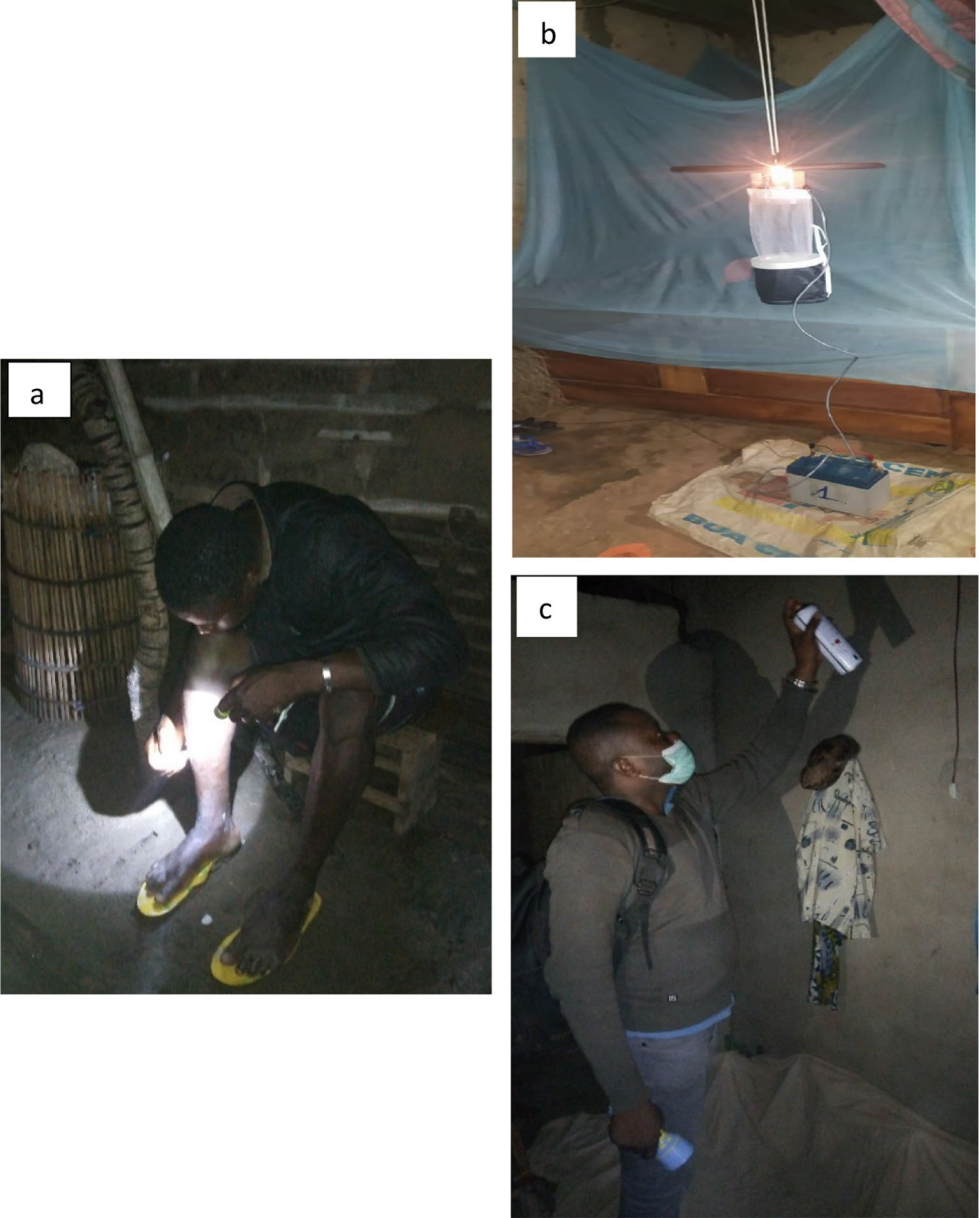
Mosquito collection methods used in the field, (**a**) Human Landing catch, (**b**) CDC light trap, (**c**) Pyrethrum Spray Catch.

HLCs were conducted with one trained adult volunteers positioned inside each house. The first group of volunteers collected mosquitoes from 19:00 pm to 01:00 am and were replaced by a second group of collectors from 01:00 to 07:00. Collections were done using hemolysis tubes and a flashlight to collect mosquitoes that landed on their lower limbs (Fig. 2a).

Standard miniature CDC light traps (Model 512; John W. Hock Company, Gainesville, FL.) were set from 19:00 pm to 7:00 am at the foot of an occupied bed fitted with a mosquito net (a study net or other net) positioned around 70 cm above the floor (Fig. 2b)^9^. In the morning, trapped mosquitoes were transferred to a paper cup and taken back to the laboratory.

In houses selected for PSCs, white sheets were installed to cover the floor and furniture and an insecticide aerosol combining 0.25% transfluthrin and 0.20% permethrin (trade name Rambo) was sprayed (Fig. 2c) for 15 s per room. Door and windows were shut and after fifteen minutes, all mosquitoes that had fallen on the sheets were recovered with forceps and stored in petri dishes.

All collections were performed in households located in the core area of each cluster to prevent contamination from adjacent clusters that might have received different nets.

### Mosquito processing

Mosquitoes from the three collection methods were identified to species level using the morphological identification key of Gillies and Meillon^33^.

A subset (approximately 30%) of *An. gambiae* s.l. collected with HLCs and CDC light traps were used to assess *P. falciparum* sporozoite infection by ELISA CSP on head and thoraces^34^. Sporozoite positive mosquitoes and a subset of negative mosquitoes caught by HLCs and CDC light traps were then used for molecular species identification, using the protocol described by Santolamazza et al*.*^35^

### Data analysis

The nightly indoor vector density was calculated at the household level for each of the mosquito collection methods (HLCs, PSCs, and CDC light traps), by dividing the total number of *An. gambiae* s.l. collected by the total number of collector night. The SR was determined by dividing the number of sporozoite positive mosquitoes by the total tested. The nightly entomological inoculation rate (EIR) was calculated by multiplying the mean nightly indoor vector density by the SR at the cluster level.

For comparison of mosquito density between collection methods, mixed effect generalised linear model with a negative binomial distribution was used for mosquito density, adjusting for study district (Cove, Ouinhi and Zaganando) as a fixed effect and including study arm as a fixed effect in the post-net distribution data. Cluster was included as a random effect in all models, with collection round also included as a random effect in post-net distribution models. A similar strategy was used with a logistic regression model for SR. Vector density and SR were compared between collection methods using HLCs as the reference.

For comparison of mosquito density and EIR between study arms, mixed effect generalized linear models with a negative binomial distribution, with collection round and cluster included as random effects were used. A mixed effect logistic regression model, with collection round and cluster included as random effects, was used to compare SR. Each collection method was assessed separately.

All data analyses were performed using the Stata 15.0 (Stata Corp., College Station, TX) software.

### Ethical considerations

Ethical approvals were issued by Benin's National Ethics Committee for Health Research (N°30/MS/DC/SGM/DRFMT/CNERS/SA, Approval n°6 of 04/03/2019), and the Ethics Committee of the London School of Hygiene and Tropical Medicine (16237-1), after reviewing the study protocol. All study participants gave their informed consent, prior to their involvement. Only trained collectors who were able to capture mosquitoes before they got bitten, were involved in the study. Before the beginning of the study, they were vaccinated against yellow fever, and treated when found positive for malaria infection. During the trial, care was provided to them at the local health facility whenever they had symptoms similar to those of malaria.

All methods were performed in accordance with the relevant guidelines and regulations.

## Results

### Performance comparison of collection methods

#### Number of collections

A total of 240 collection nights (4 households * 60 clusters) were performed with each collection method at baseline between September and October 2019. Post-net distribution, PSCs and HLCs were conducted eight times over two years in each cluster for a total of 1920 collection nights per method, while CDC light traps were performed twice (480 collection nights) in all clusters at 16 months (June–July 2021) and 19 months (September–October 2021) post-net distribution.

#### Mosquito species composition

**Figure 3:**
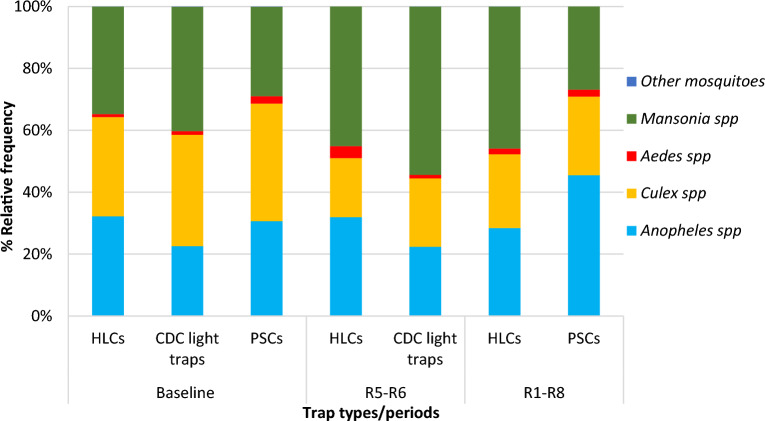
Mosquito species composition collected by HLCs, CDC light traps and PSCs in the study area. R5-R6: Round 5 to 6 (June-July 2021 and September–October 2021: 16th to 19th month post-net distribution), R1-R8: Round 1 to 8 (June 2020 to April 2022: whole post-net distribution period).

*Anopheles* mosquitoes comprised 32.2%, 22.6%, and 30.6% of all mosquitoes caught at baseline for HLCs, CDC light traps, and PSCs, respectively, with the vast majority being *An. gambiae* s.l. (88% in all collection methods). Other *Anopheles* species caught in smaller proportions by all three collection methods were *An. funestus* gr, *An. nili* gr, *An. ziemmani*, and *An. brohieri.* Over the whole post-net distribution period (June 2020–April 2022), the proportion of *Anopheles* mosquitoes (of all mosquitoes caught) was 28.4% for HLCs, and 45.5% for PSCs. In the two post-net distribution rounds where both HLCs and CDC light traps were used, *Anopheles* mosquitoes accounted for 32.0% of HLC caught mosquitoes and 22.4% of CDC light trap caught mosquitoes. Overall, HLCs and PSCs collected a higher proportion of *Anopheles* mosquitoes as compared to CDC light traps (Fig. 3).

**Figure 4:**
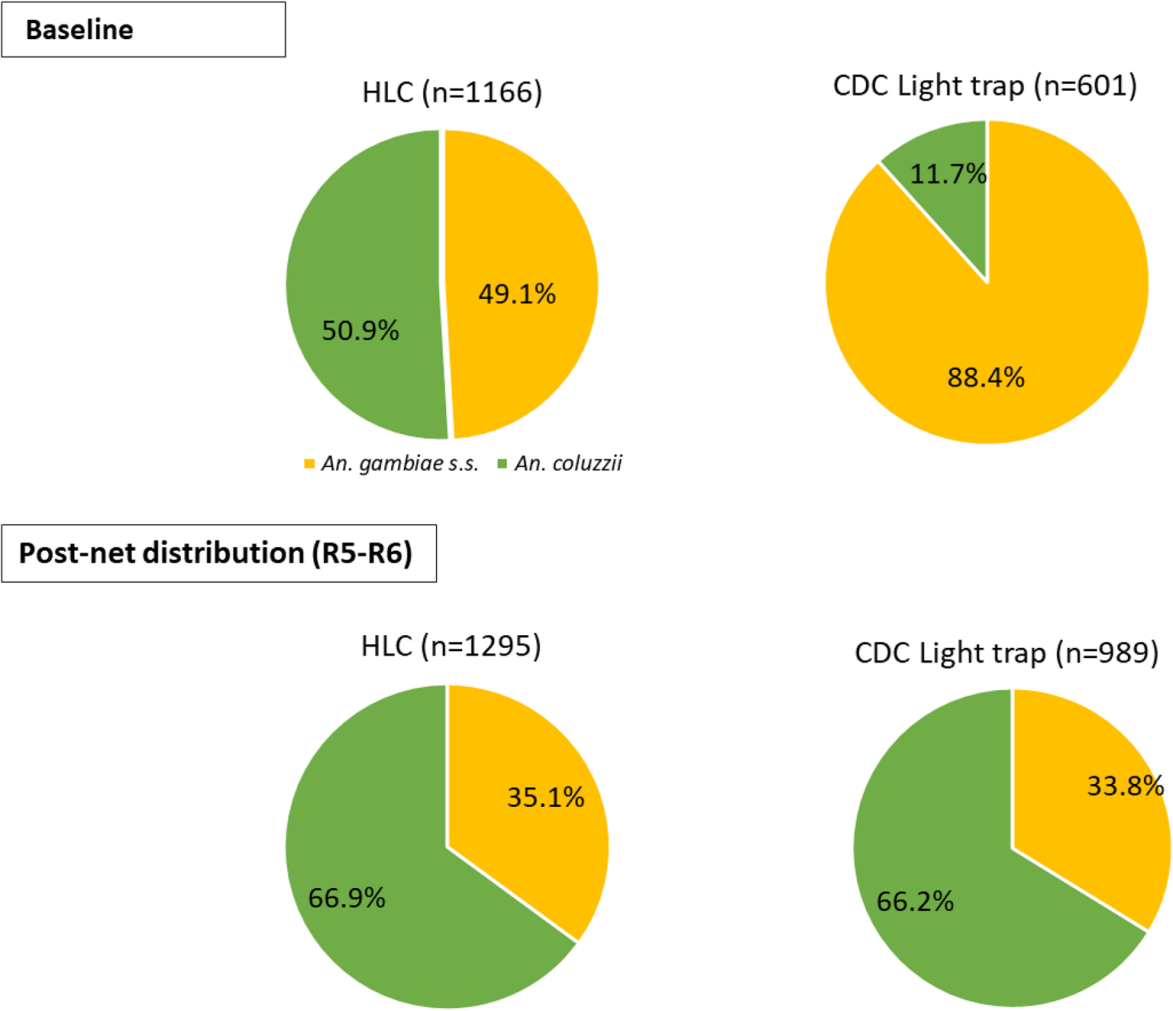
Proportion of *An. gambiae* s.l.* (*collected through HLCs and CDC light traps at baseline, and post-net distribution) identified to molecular species level. R5-R6: Rounds 5 and 6 (June-July 2021 and September–October 2021: 16th to 19th month post-net distribution), R1-R8: Round 1 to 8 (June 2020 to April 2022: whole post-net distribution period).

At baseline, a higher proportion of *An. coluzzii* were caught with HLCs (50.9%) than with CDC light traps (11.7%). Post-net distribution, *An. coluzzii* formed two thirds of the total tested mosquitoes for both HLCs and CDC light traps (66% for both collection methods with pooled data: cluster range for HLCs (0.7–98.1%); cluster range for CDC light traps (0–100%). The rest of the *Anopheles* species tested were *An. gambiae* s.s. for both baseline and post-net distribution periods (Fig. 4).

At baseline, in all three collection methods, at least one third of mosquitoes caught were *Culex* spp. (between 32.1 and 38.0%). The proportion of *Culex* caught was reduced post-net distribution in all three collection methods, to around a quarter of the collection [23.8% for HLCs, 25.4% for PSCs, and 22.1% for CDC light traps* (*in two rounds only)] (Fig. 3).

At baseline, *Mansonia* spp accounted for 34.7%, 40.2%, and 28.8% of each collection for HLCs, CDC light traps and PSCs, respectively. Post-net distribution, the proportion of *Mansonia* increased for HLCs (45.1%) and CDC light traps (54.4%) (in two rounds only) but remained similar to baseline for PSCs (26.9%). Overall, *Mansonia* mosquitoes were more likely to be caught by HLCs and CDC light traps compared to PSCs (Fig. 3).

Overall, *Aedes* spp. were sampled by the three collection methods at low frequencies (< 4%). *Aedes* spp. was mostly collected through HLCs and PSCs. Other mosquitoes caught in very lower proportions (< 0.07%) included *Coquillettidia* and *Eretmapodites*.

#### Density and SR of An. gambiae s.l collected at baseline and post-net distribution through HLCs, CDC light traps, and PSCs

A total of 10,093 specimens of *An. gambiae* s.l. were collected at baseline and 11,465 post-net distribution with all collection methods. At baseline, the mean indoor density of *An. gambiae* s.l. was significantly higher with HLCs [26.6/person/night] compared with CDC light traps [13.0/trap/night; Density Ratio = 0.4 (95% CI 0.33–0.48), *p* < 0.001], and PSCs [2.5/house/day; DR = 0.07 (95% CI 0.06–0.08); *p* < 0.001]. A similar trend was observed post-net distribution with a mean indoor vector density of 15.9/person/night for HLCs, compared to 6.8/trap/night [DR = 0.36 (95% 0.30–0.42), *p* < 0.001] for CDC light traps, and 1.1/house/day [DR = 0.06 (95% CI 0.05–0.07); *p* < 0.001] for PSCs (Table 1).

**Table 1:** Density and SR of *An. gambiae* s.l collected at baseline and post-net distribution (rounds 5 and 6) through HLCs, CDC light traps* (*density only), and PSCs.

Periods	Collection methods	Number of collections	*N of An*	Density (b/p/n)	aDR	95%CI	p-value	N of *An* (Tested)	N of *An* (Positive)	SR (%)	aOR	95%CI	*p*-value
Baseline	HLC	240	6373	26.6	1 (Ref)			2264	66	2.9	1 (Ref)		
	CDC light trap	240	3125	13.0	0.4	0.33–0.48	< 0.001	2453	50	2.0	0.65	0.46–0.93	0.0184
	PSC	240	593	2.5	0.07	0.06–0.08	< 0.001	n/a	n/a	n/a	n/a	n/a	n/a
Post-net distribution	HLC	480	7669	15.9	1 (Ref)			2819	62	2.2	1 (Ref)		
	CDC light trap	480	3273	6.8	0.36	0.30–0.42	< 0.001	1629	23	1.4	0.65	0.41–1.0	0.0544
	PSC	480	523	1.1	0.06	0.05–0.07	< 0.001	n/a	n/a	n/a	n/a	n/a	n/a

N of *An*, number of *An. gambiae* s.l.; IRR, incidence rate ratio; CI, confidence interval; SR, sporozoite rate; aOR, adjusted odd ratio; n/a, no data; b/p/n, bite/person/night.

Density and SR were adjusted for study area for baseline and post-net distribution. Study arm included as a fixed effect in the post-net distribution model. Cluster was included as a random effect in all models. Collection round was included as a second random effect in the post-net distribution model.

Overall, the SR in *An. gambiae* s.l. collected in HLCs was slightly higher compared to those in CDC light traps. At baseline it was 2.9% for HLCs and 2% for CDC light traps [aOR = 0.65 (95% CI 0.46–0.93, *p* = 0.0184)], and 2.1% with HLCs, compared to 1.4% with CDC light traps [aOR = 0.65 (95% CI 0.41–1.0); (*p* = 0.0544)] during post-intervention (Table 1).

### Performance of different collection methods to estimate the impact of the interventions on entomological indicators

#### Performance of HLCs and PSCs to estimate the impact of pyriproxyfen-pyrethroid LLINs and chlorfenapyr-pyrethroid LLINs on the density of An. gambiae s.l

The average number of *An. gambiae* s.l. collected was 10–15 times lower with PSCs compared to HLCs (Table 1). However, the reduction in density observed in mosquitoes caught using PSCs between each of the intervention arms compared to the control arm was of similar magnitude as the HLCs (Table 2).

**Table 2:** Density per arm of *An. gambiae* s.l. collected through PSCs and HLCs at years 1 and 2 post-net distribution, and overall.

Arms	PSCs: resting density (*An*/h/d)					HLCs: biting density (b/p/n)			
	N of An	N of HH	Mean (SD)	DR (95% CI)	*p*-value	N of An	Mean (SD)	DR (95% CI)	*p*-value
Year 1									
Std LLIN	470	320	1.46 (2.64)	1 (Ref)		7313	22.85 (35.77)	1 (Ref)	
PPF LLIN	249	320	0.77 (1.79)	0.44 (0.24–0.79)	0.0062	4490	14.03 (27.81)	0.51 (0.26–0.99)	0.0472
CFP LLIN	206	320	0.64 (1.27)	0.44 (0.24–0.79)	0.0060	3500	10.94 (17.14)	0.47 (0.24–0.92)	0.0274
Year 2									
Std LLIN	641	320	2 (6.65)	1 (Ref)		7278	22.74 (32.23)	1 (Ref)	
PPF LLIN	320	320	1 (2.44)	0.59 (0.31–1.1)	0.0975	4093	12.79 (17.78)	0.68 (0.32–1.46)	0.3326
CFP LLIN	272	320	0.85 (2.95)	0.42 (0.22–0.8)	0.0084	2809	8.77 (11.92)	0.41 (0.19–0.86)	0.0192
Overall									
Std LLIN	1111	640	1.73 (5.01)	1 (Ref)		14,591	22.79 (34.05)	1 (Ref)	
PPF LLIN	569	640	0.89 (2.14)	0.51 (0.31–0.84)	0.0095	8583	13.41 (23.35)	0.59 (0.3 -1.17)	0.1368
CFP LLIN	478	640	0.75 (2.27)	0.43 (0.26–0.72)	0.0014	6309	9.85 (14.8)	0.44 (0.22- 0.86)	0.0177

PSC, Pyrethrum Spray Catch; HLC, Human Landing Catch; N of *An*, Number of *An. gambiae* s.l.; N of HH, Number of Household; SD, Standard Deviation; DR, Density Ratio; Std LLIN, Standard LLIN; PPF LLIN, Pyriproxyfen-pyrethroid- LLIN; CFP LLIN, Chlorfenapyr-pyrethroid- LLIN; b/p/n, bite/person/night; An/h/d (number of Anopheles gambiae s.l./house/day).

Overall, there was a 49% reduction in *An. gambiae* s.l. density in the pyriproxyfen-pyrethroid LLIN arm [DR = 0.51 95% CI (0.31–0.84); *p* = 0.0095] and 57% in the chlorfenapyr-pyrethroid LLIN arm [DR = 0.43 95% CI (0.26–0.72); *p* = 0.0014], compared to standard pyrethroid-only LLIN arms with PSCs, while the reduction was 41% [DR = 0.59 95% CI (0.30–1.17); *p* = 0.1368] in the pyriproxyfen-pyrethroid LLIN arm and 56% [DR = 0.44 95% CI (0.22–0.86); *p* = 0.0177] in chlorfenapyr-pyrethroid LLIN arm with HLCs.

Overall, the reduction in *An. gambiae* s.l. was significant using PSCs in the pyriproxyfen-pyrethroid LLIN arm. This effect was particularly clear in the first year of the study. Overall, there was no strong evidence for a reduction in mosquito density in the pyriproxyfen-pyrethroid LLIN arm using HLCs. Similar conclusions regarding the efficacy of the chlorfenapyr-pyrethroid LLIN arm could be made for both HLCs and PSCs, with a much more pronounced reduction in vector density in the chlorfenapyr-pyrethroid LLIN arm, compared to the pyrethroid-only LLIN arm, irrespective of the mosquito collection method used. This was observed during the first and second year of the trial, with both HLCs and PSCs (Table 2).

#### Entomological impact of pyriproxyfen-pyrethroid LLINs and chlorfenapyr-pyrethroid LLINs compared to standard pyrethroid-only LLINs on vector density, SR and EIR using HLCs or CDC light traps at round 5 and 6 post-net distribution

With HLCs, there was weak evidence for a 41% reduction in mean indoor density of *An. gambiae* s.l. in the chlorfenapyr-pyrethroid LLIN arm [DR = 0.59 95% CI (0.33–1.05); *p* = 0.0776], while no clear reduction was observed in the pyriproxyfen-pyrethroid LLIN arm [DR = 0.95 95% CI (0.53–1.70); *p* = 0.8829], compared to the standard pyrethroid-only LLIN arm. A similar trend was observed with the CDC light traps, with some evidence for a 53% reduction in mean indoor vector density in the chlorfenapyr-pyrethroid LLIN arm [DR = 0.47 95% CI (0.22–1.0); *p* = 0.0517], and no reduction in the pyriproxyfen-pyrethroid LLIN arm [DR = 1.12 95% CI (0.53–2.36); *p* = 0.7478], compared to the standard pyrethroid-only LLIN arm (Table 3).

**Table 3:** Efficacy of pyriproxyfen-pyrethroid LLINs and chlorfenapyr-pyrethroid LLINs compared to Std LLINs through the evaluation of Vector Density, SR and EIR using both HLCs and CDC light traps at round 5 and 6 post-net distribution.

Collection methods and arms	Density per night per household					SR				EIR per night per cluster*		
	N of *An*	N of trap/person night	Mean (SD)	DR (95% CI)	*p*-value	n/N (%)	95% CI	OR (95% CI)	*p*-value	Mean (SD)	DR (95% CI)	*p*-value
CDC light trap												
Std LLIN	1267	160	7.91 (15.81)	1 (Ref)		8/627 (1.27)	0.6–2.44	1 (Ref)		0.11 (0.32)	1 (Ref)	
PPF LLIN	1373	160	8.58 (15)	1.12 (0.53–2.36)	0.7478	12/634 (1.89)	1.08–3.28	1.49 (0.6—3.67)	0.3837	0.17 (0.42)	1.52 (0.47–4.93)	0.4832
CFP LLIN	633	160	3.95 (9.31)	0.47 (0.22–1)	0.0517	3/368 (0.81)	0.26–2.44	0.64 (0.16 -2.41)	0.5058	0.03 (0.11)	0.24 (0.03–1.91)	0.1797
HLC												
Std LLIN	3322	160	20.76 (24.7)	1 (Ref)		22/983 (2.23)	1.5–3.31	1 (Ref)		0.52 (1.10)	1 (Ref)	
PPF LLIN	2533	160	15.8 (18.14)	0.95 (0.53–1.7)	0.8829	24/1017 (2.35)	1.47–3.75	1.05 (0.56–1.97)	0.8717	0.27 (0.58)	0.56 (0.23–1.35)	0.2008
CFP LLIN	1814	160	11.3 (12.73)	0.59 (0.33–1.05)	0.0776	16/819 (1.95)	1.11–3.40	0.86 (0.43–1.73)	0.6917	0.19 (0.39)	0.40 (0.15–1.05)	0.0624

SR, sporozoite rate; EIR, Entomological Inoculation Rate; N of *An*, Number of *An. gambiae* s.l.; SD, standard deviation; DR, Density Ratio; n, Number of positive *An. gambiae* s.l.; N, Number of Tested *An. gambiae* s.l.; CI, Confidence Interval; OR, Odd Ratio; Std LLIN, standard pyrethroid-only LLIN; PPF LLIN, pyriproxyfen-pyrethroid LLIN; CFP LLIN, chlorfenapyr-pyrethroid LLIN.

With HLCs, there was no strong evidence for a reduction in SR in either the chlorfenapyr-pyrethroid LLIN arm [OR = 0.86 95% CI (0.43–1.73); *p* = 0.6917], or the pyriproxyfen-pyrethroid LLIN arm [OR = 1.05 95% CI (0.56–1.97); *p* = 0.8717], compared to the standard pyrethroid-only LLIN arm. With CDC light traps, the trend was similar [OR = 0.65 95% CI (0.16–2.41); *p* = 0.5058 in the chlorfenapyr-pyrethroid LLIN arm, and OR = 1.49 95% CI (0.6–3.67); *p* = 0.3837 in the pyriproxyfen-pyrethroid LLIN arm, relative to the standard pyrethroid-only LLIN arm] (Table 3).

Similarly, there was no strong evidence for reductions in EIR for the chlorfenapyr-pyrethroid LLIN arm [OR = 0.56 95% CI (0.23–1.35); *p* = 0.2008] and the pyriproxyfen-pyrethroid arm [OR = 0.40 95% CI (0.15–1.05); *p* = 0.0624], compared to the standard pyrethroid-only LLIN arm. The same trend was seen with CDC light traps (Table 3).

## Discussion

The comparison of three mosquito collection methods (HLCs, CDC light traps and PSCs) as part of a large RCT testing dual-a.i. LLINs in Benin showed that HLCs resulted in far greater numbers of mosquitoes being collected as compared to CDC light traps and PSCs. Overall, the mean density of *An. gambiae* s.l. collected was half with CDC light traps compared to HLCs, while it was 10 times lower with PSCs. However, despite this difference, CDC light traps and PSCs often measured similar reductions in mosquito density, SR and EIR* (*CDC light traps only) as compared to HLCs between study arms.

The higher *Anopheles* densities caught with HLCs compared to CDC light traps in the present study has been also reported in several other studies^10,17^. In those studies HLCs caught 1.5 to 1.7 times more *Anopheles* vectors than CDC light traps; in the present study HLCs caught 2.2 (95% CI 2.1–2.3) times more (Table 1). This shows there was no consistent difference in density between HLCs and CDC light traps, as previously found by Briët et al.^36^ after reviewing 13 studies. One of the possible reasons for the higher vector densities with HLCs compared to CDC light traps, could be due to the fact that numerous stimuli (olfactory, visual cues, volatiles, body heat, and humidity) involved in the collections using human bait exert a strong attraction on host-seeking mosquitoes, while only visual ones are used during CDC light trap collections. Moreover, the study LLINs which sleepers were under and which were installed close to the CDC light traps might have repelled some of the host seeking mosquitoes, so reducing their density as previously observed by Kirby et al*.*^37^.

In the present study, PSCs resulted in a far lower (at least 10 (95% CI 9.8–11.7) times) mean indoor vector density compared to HLCs (Table 1), as previously observed by Ndiath et al.^10^ Originally, this method was used to sample mosquitoes that rest inside houses and therefore cannot be considered a quantitative method for evaluating indoor vector density, as mosquitoes might have left the houses early in the morning to seek for outdoor hosts due to unsuccessful blood meal intake that occurred overnight. The latter observation might be due to a possible repellent effect induced by the insecticides incorporated in the trial LLINs that also constituted a physical barrier that reduced the host-vector contact as previously observed by Sovi et al.^38^ In addition, in the Cove, Ouinhi and Zagnanando districts where malaria vectors were highly pyrethroid-resistant with several resistance mechanisms^31^, it is possible that the aerosol insecticide used for the PSCs failed to knock down or kill all resting mosquitoes, resulting in a lower density of collected samples. Finally, the lack of standardization (different collection times from cluster to cluster, possible dispersion of mosquitoes in the presence of eaves or holes in the walls) for this method could also have had an impact on mosquito density caught using PSCs.

Conflicting results regarding SR have been found in previous studies with some finding SR was similar for both HLCs and CDC light traps^10,17^ whilst others, found it was higher in CDC light traps compared to HLCs^16,39^. However, none of these trends was observed in the present study as the SR was 1.5 (95% CI 1.4–1.6) times higher with HLCs than with CDC light traps at both baseline and post-net distribution (Table 1). This might be due to the relative time in the physiological age structure of the vector populations, as collections occurred only once at baseline, and twice post-net distribution.

Both CDC light traps and HLCs were used to assess the effect of chlorfenapyr-pyrethroid and pyriproxyfen-pyrethroid LLINs on key entomological indicators (density, SR, and EIR) of malaria transmission as compared to pyrethroid-only net. Overall, the two collection methods showed that the chlorfenapyr-pyrethroid LLIN tended to be more effective than the pyriproxyfen-pyrethroid LLIN, but with weak evidence, as there were only two rounds of collection (Table 3). This finding is reminiscent of the large RCT conducted by Mosha et al*.*^40^ in Tanzania, using CDC light traps, that showed superior efficacy of chlorphenapyr-pyrethroid LLINs on vector density and EIR, which was not observed for pyriproxyfen-pyrethroid LLINs.

PSCs and HLCs showed a significant reduction in vector density in the chlorfenapyr-pyrethroid LLIN arm. By comparison, PSCs displayed a significant reduction in vector density in the pyriproxyfen-pyrethroid LLINs arm, while HLCs did not (Table 2). This might be because PSCs caught a higher relative frequency of *An. gambiae* s.l. as compared to HLCs (44.6% vs 27.2%). This made it easier to see differential impact of the two dual-a.i. LLINs with PSCs, as compared with HLCs.

Overall, the same morphologically identified mosquito genera were collected using the three collection methods. The proportion of *Culex* mosquitoes decreased post-net distribution, compared to baseline in all collection methods. Post-net distribution, the proportion of *Mansonia* spp. increased in HLCs and CDC light traps but remained the same in PSCs as compared to baseline. For *Anopheles* mosquitoes, their proportion remained similar in HLCs and CDC light traps and increased in PSCs post-net distribution, as compared to baseline (Fig. 3). For the three mosquito genera, the changes in species composition observed post-net distribution in certain collection methods compared to baseline could be due to the seasonality. Indeed, the baseline collection occurred at only one time point (short rainy season: September–October 2019), so could not capture a representative image of the species composition compared to the post-net distribution period which occurred over 8 time points (all seasons: June 2020 to April 2022). From baseline to the post-net distribution period, changes in *Mansonia* and *Anopheles* species composition followed the same trend in HLCs and CDC light traps, while it did not in PSCs. This might be because both HLCs and CDC light traps collect host seeking mosquitoes^41^, while PSCs sample indoor resting ones^10^.

At the molecular species level, the composition was similar post-intervention for HLCs and CDC light traps as observed by Mawejje et al.^17^ However, relative molecular species composition was different between HLCs and CDC light traps at baseline, with *An. gambiae* s.s. mostly collected through CDC light traps, and in similar proportions to *An. coluzzii* through HLCs (Fig. 4). This might be due to the difference in how sampled houses for both collection methods have been selected between baseline and post-net distribution period. The decline in the proportion of *An. gambiae* s.s. observed post-net distribution compared to baseline for both HLCs and CDC light traps (Fig. 4), suggests that the intervention tools implemented were more effective on this mosquito species, which was not the case for *An. coluzzii*.

Limitations for the present study include the only two post-net distribution rounds of data collection performed with CDC light traps, the lack of molecular (species identification, and *P. falciparum* sporozoite infection) data for mosquito collected through PSCs, the non-inclusion of environmental conditions (temperature, humidity) as fixed effects in the analyses to account for overtime variations, as well as the fact that the trial was conducted in a single region, which limits the generalization of the findings.

To our knowledge, the present study is the first one to evaluate whether PSCs and CDC light traps are equally as effective as HLCs in estimating the differential impact of dual-a.i. LLINs on key entomological indicators of malaria transmission. Overall, HLCs collected more mosquito vectors than CDC light traps and PSCs, so remain the gold standard method to be used for entomological monitoring, notwithstanding the ethical issues involved and the fact that in the daily life, nobody in the community is going to sit and expose their lower limbs to receive potential mosquito bites overnight—so the mosquitoes caught might not be reflective of a realistic risk of biting. However, CDC light traps and PSCs were found to be as effective as HLCs in evaluating the differential impact of dual-a.i. LLINs on key entomological indicators of malaria transmission as compared to pyrethroid-only nets.

## Conclusion

In our study area, HLCs may be prioritized for single cross-sectional studies, as this collection method tends to collect more mosquitoes. However, due to the ethical issues raised by this collection method, CDC light trap which is a considerably simpler, cheaper and logistically easier method that also collected host-seeking mosquitoes, could be used as an alternative option, as part of longitudinal entomological studies given it showed potential in assessing the differential impact of next-generation vector control tools.

PSC that collected a much lower number of indoor resting vectors, was also able to evaluate the efficacy of next-generation nets compared to standard pyrethroid-only nets.

## Data Availability

The datasets generated and/or analysed during the present study are available on reasonable request from the corresponding authors.
